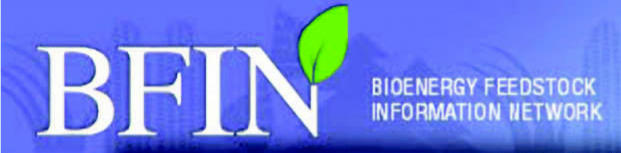# EHPnet: Bioenergy Feedstock Information Network

**Published:** 2007-02

**Authors:** Erin E. Dooley

Oak Ridge National Laboratory hosts a web-based resource center, the Bioenergy Feedstock Information Network, that assembles a wealth of information from the Department of Energy, the laboratory itself, Idaho National Laboratory, the National Renewable Energy Laboratory, and other research organizations working on alternative fuels. The site, **http://bioenergy.ornl.gov/**, can be navigated using either the top toolbar, which features information grouped by seven topics, or the links down the left side of the homepage, which group resources by type (some browsers do not allow use of the top toolbar).

The Biomass Basics topic from the toolbar offers a collection of fact sheets, journal articles, weblinks, presentations, and reports that provide general insight into the biofuel production industry. One of the fact sheets, “The Bioenergy Cycle: A Vision of the Future,” details how the bioenergy cycle works in a best-case scenario. The Economics selection in the tool bar has much more information in this vein, with a number of presentations on the topic that have been developed and presented by collaborating members. One presentation discusses how bioenergy crop production might impact the U.S. agricultural sector, including effects on crop prices and changes in land area allotted for crops.

Impacts of bioenergy production on air, biodiversity, soil, water, and other areas are subtopics within the Environment section of the network website. Among the items in the Environment section page is a document titled “Energy Crops and the Environment,” which looks at how growing crops for energy purposes can positively impact the environment by improving water quality and decreasing erosion and runoff. Other reports in the Water subsection of the Environment section address environmental benefits of cellulosic energy crops (such as switchgrass) and soil and water quality aspects of herbaceous and woody crop production.

Within the Biomass Resources section of the website are several other reports, databases, weblinks, presentations, and fact sheets that address environmental issues. One is a National Audubon Society report, *An Analysis of the Environmental Impacts of Energy Crops in the USA*, which explores the tipping point at which biomass crop cultivation would begin to negatively affect the environment. Other reports examine research priorities for energy crop production and ways of ensuring the sustainability of feedstock production.

The Supply System portion of the site offers information on the various stages of biofuel production, including harvesting, preprocessing, storage, systems integration, and transportation. Finally, the Glossary section lists not only terms used in the bioenergy industry, but also a seven-section assortment of Frequently Asked Questions on topics ranging from the basic to the more technical.

## Figures and Tables

**Figure:**